# Mechanisms and functions of intestinal vascular specialization

**DOI:** 10.1084/jem.20222008

**Published:** 2023-12-05

**Authors:** Jeremiah Bernier-Latmani, Alejandra González-Loyola, Tatiana V. Petrova

**Affiliations:** 1 Department of Oncology, University of Lausanne and Ludwig Institute for Cancer Research Lausanne, Lausanne, Switzerland; 2 Aragon Health Research Institute (IIS Aragón), Zaragoza, Spain; 3 Swiss Institute for Experimental Cancer Research, School of Life Sciences, École polytechnique fédérale de Lausanne, Lausanne, Switzerland

## Abstract

The intestinal vasculature has been studied for the last 100 years, and its essential role in absorbing and distributing ingested nutrients is well known. Recently, fascinating new insights into the organization, molecular mechanisms, and functions of intestinal vessels have emerged. These include maintenance of intestinal epithelial cell function, coping with microbiota-induced inflammatory pressure, recruiting gut-specific immune cells, and crosstalk with other organs. Intestinal function is also regulated at the systemic and cellular levels, such that the postprandial hyperemic response can direct up to 30% of systemic blood to gut vessels, while micron-sized endothelial cell fenestrations are necessary for nutrient uptake. In this review, we will highlight past discoveries made about intestinal vasculature in the context of new findings of molecular mechanisms underpinning gut function. Such comprehensive understanding of the system will pave the way to breakthroughs in nutrient uptake optimization, drug delivery efficiency, and treatment of human diseases.

## Teaser

This review highlights classic and recent findings detailing molecular mechanisms of organ-specific functions of blood and lymphatic intestinal vessels, including nutrient absorption, epithelial cell maintenance, and immune cell recruitment. It also proposes future research directions for the field.

## Introduction

The gastrointestinal (GI) tract is a portal for the body’s interaction with the outside world. It must serve to distribute nutrients systemically while simultaneously maintaining a barrier to prevent infection from gut-borne microorganisms. Given this crucial role for systemic health, the intestine has been a target of widespread research that revealed gut specialization in the epithelial, immune, nervous, muscle, and fibroblast systems (Allaire et al., 2018; Brügger and Basler, 2023; Gehart and Clevers, 2019; Mowat and Agace, 2014; Sylvestre et al., 2023; Yoo and Mazmanian, 2017).

This specialization also extends to the intestinal vascular system, which displays unique features and crosstalk with other gut cell types. In addition to systemic nutrient absorption, recent work shows a role for vessels in promoting gut homeostasis independent of food uptake through interaction with other intestine-resident cell types. Therefore, this review will summarize the mechanistic knowledge of intestinal vascular biology while also positing new directions for the field.

## Intestinal organization

### Crypt/villus axis

**Figure 1.**
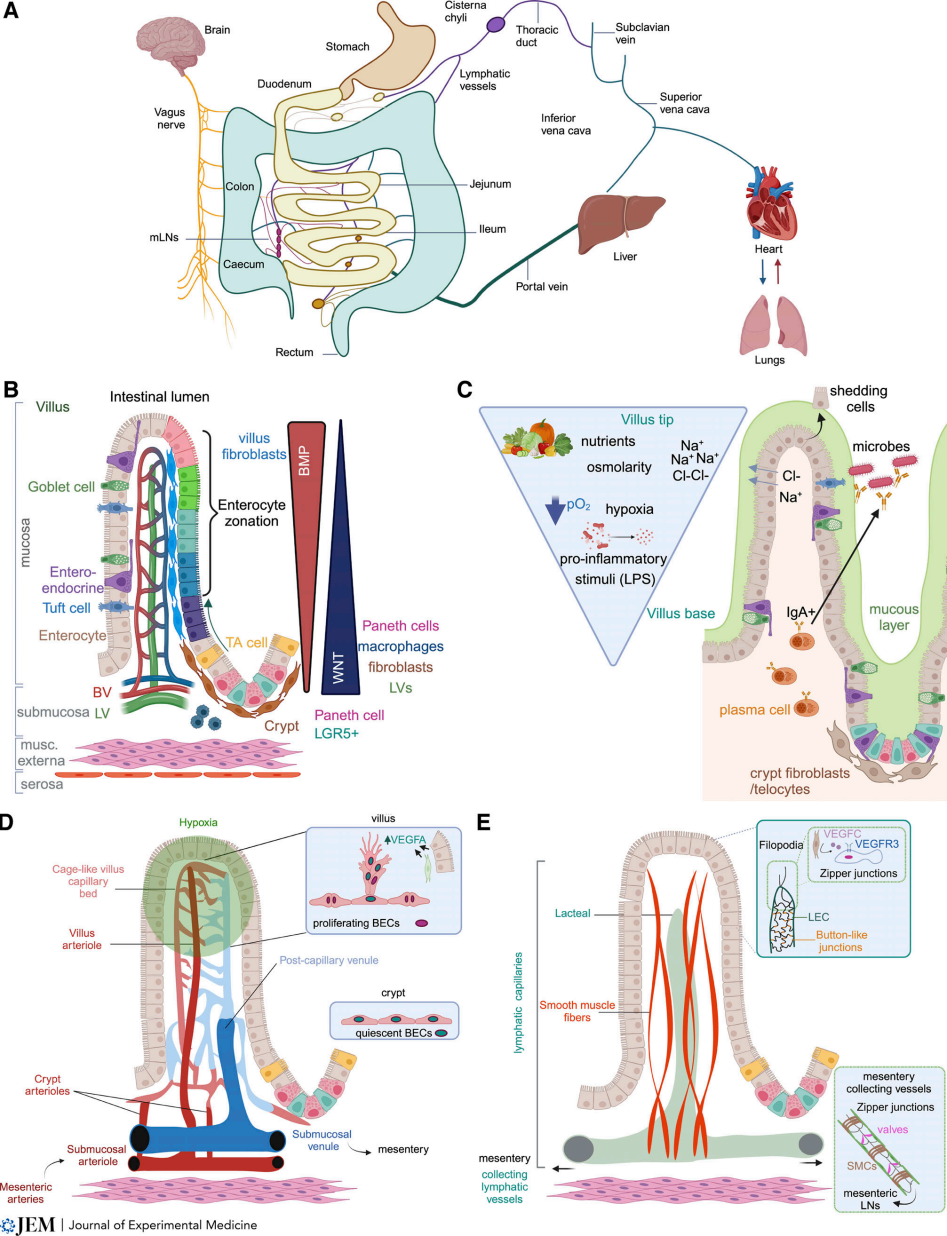
**Organization of the GI tract and gut vasculature. (A)** GI tract organization and contribution to systemic circulation. All intestine-derived blood flows through the portal vein and into the liver. Intestinal lymph drains to the MLNs before entering systemic blood circulation via the cisterna chyli and thoracic duct. The gut-to-brain connection through the nervous system is also depicted. **(B)** Epithelial cells are maintained by constant proliferation of crypt-housed stem cells, which differentiate upon migration to the villus to give rise to all differentiated cell types. Gradients of Wnt and Bmp ligands along the villus/crypt axis promote epithelial stemness and differentiation, respectively. BV, blood vessels; LV, lymphatic vessels. Enterocyte zonation is marked along villus axis. **(C)** Environmental factors such as nutrients, osmolarity, oxygen pressure, and microbiota-derived inflammatory molecules also form gradients along the villus/crypt axis. **(D)** Organization of the blood vasculature along the villus/crypt axis. Villus blood capillaries are VEGFA dependent (due to villus tip hypoxia) and display signs of angiogenesis (filopodia), while crypt capillaries reside in normoxic conditions and are VEGFA independent (BECs, blood endothelial cells). **(E)** Organization of the lymphatic vasculature along the villus/crypt axis. Villus-housed blind-ended lymphatic capillaries (lacteals) display signs of lymphangiogenesis such as filopodia and LEC proliferation and are ensheathed by villus SMCs. Crypt lymphatic capillaries do not display filopodia and crypt LECs rarely proliferate. All intestinal lymphatic capillaries, except for those with filopodia, display permeable button cell–cell junctions distinguishing them from mesenteric lymphatic collecting vessels, which display zipper cell–cell junctions, valves, and SMC coverage.

The intestinal tract is a hollow tube lined by epithelial cells from the trachea to the anus allowing a route to extract nutrients and water from ingested food (Fig. 1 A). After mechanical and chemical digestion in the upper GI tract, nutrients are absorbed by the small intestine, and this review will mostly focus on this segment. The distinctive small intestinal cellular architecture consists of finger-like projections, called villi, which protrude into the intestinal lumen and are separated by troughs in the tissue called crypts. A single layer of epithelial cells overlays distinct villus- and crypt-associated (also called submucosal) stromal cells. The submucosa is sheathed in a muscle cell–rich layer, which itself is covered in a single layer of mesothelial cells (Fig. 1 B).

The crypt/villus axis is highly zonated with cellular, cell signaling, and physicochemical gradients necessary for maintaining intestinal architecture and function. The essential function of epithelial maintenance is performed through continuous regeneration from proliferating *Lgr5*^+^ epithelial intestinal stem cells (ISCs) which reside in the bottom of crypts (Gehart and Clevers, 2019). Wnt signaling is necessary for maintenance of these cells; therefore, the crypt niche maintains several overlapping layers of Wnt ligand and Wnt signaling potentiator sources including crypt resident Paneth cells, pericryptal fibroblasts, macrophages, and lymphatic vessels. In addition to stem and Paneth cells, the crypt contains proliferating transient amplifying cells that are progenitors for the differentiated cells lining the villus (Fig. 1 B; Brügger and Basler, 2023; Gehart and Clevers, 2019; Sylvestre et al., 2023).

While the crypt provides continuous regeneration of the epithelium, the finger-like villi project into the lumen, increasing surface area to carry out the critical role of nutrient absorption. Epithelial cells are constantly migrating from the crypts toward villi while differentiating to become absorptive enterocytes, chemosensitive and signaling enteroendocrine and tuft cells, and mucus-producing goblet cells (van der Flier and Clevers, 2009). In contrast to crypt-associated Wnt signaling, villi display high Bmp signaling (Fig. 1 B). Villus fibroblasts are the source of Bmp ligands that promote epithelial differentiation and maturation (Brügger and Basler, 2023; Felsenthal and Vignjevic, 2022; Sylvestre et al., 2023). Differentiated villus enterocytes are not homogeneous; instead, they are zonated along the proximal-to-distal villus axis and exhibit distinct expression of nutrient transporters and channels (Fig. 1 B; Moor et al., 2018). Bmp signaling regulates a lipid-handling program of intestinal villus tip enterocytes and enforces villus epithelial cell zonation through the transcription factor MAF (Bara et al., 2022; Beumer et al., 2022; Cosovanu et al., 2022; González-Loyola et al., 2021). Therefore, villus and crypt epithelial cells and fibroblasts are cosegregated in the villus and crypt zones through multiple levels of signaling.

Villi display inherent structural peculiarities in addition to being swathed by a pro-inflammatory luminal milieu, giving them distinct physicochemical attributes. Intestinal villus tips are the site of constant epithelial cell death (Williams et al., 2015) and levels of incoming nutrients, osmolarity, hypoxia, and proinflammatory stimuli (e.g., LPS) are all higher at the distal villus tip compared with the proximal villus base (Fig. 1 C; Alpers, 1972; Hallbäck et al., 1978; Hallbäck et al., 1991; Kinter and Wilson, 1965; Parker et al., 2019; Sjöqvist and Beeuwkes, 1990; Williams et al., 2013). Therefore, villus cellular patterning and signaling are adapted to this distinct microenvironment. For example, subepithelial fibroblasts (“telocytes”) enhance barrier function through interaction with both epithelial and stromal cells, and villus smooth muscle cells (SMCs) contract villi to promote nutrient uptake (Brügger and Basler, 2023; Felsenthal and Vignjevic, 2022; Sylvestre et al., 2023). In addition, intestinal-specific immune cells reside in villi, including IgA-producing plasma cells, to monitor and restrict intestinal microbiota-generated inflammation (Fig. 1 C; Mowat and Agace, 2014).

The small intestine is divided into three sub-compartments: the duodenum, jejunum, and ileum, and villus size and function are distinct among these regions (Fig. 1 A). The duodenum and upper jejunum are just downstream of the stomach and are the primary sites of nutrient extraction from the lumen. In line with this function, they harbor larger villi than the distal zones (Bernier-Latmani and Petrova, 2017). Nutrient availability directly controls villus size, as starvation and refeeding lead to duodenal villus atrophy or regrowth, respectively (Altmann, 1972). Although the mechanisms are not entirely clear, villus size is inversely correlated to the amount of microbiota present. Germ-free or antibiotics-treated mice display larger villi than normally raised mice, and villi are smaller in the ileum, which harbors a higher microbial load compared to the upper small intestine (Smith et al., 2007). Microbiota and intestinal eosinophils contribute to villus size maintenance. Eosinophil-proficient mice, raised in the presence of microbiota, display larger villi than eosinophil-deficient mice; however, villi are larger in germ-free mice regardless of eosinophil status (Ignacio et al., 2022). In addition to the microbiota, other gut lumen contents also differ among the small intestinal zones. Villi in the upper small intestine are exposed to higher levels of ingested food and bile acids, which promote fat metabolism and absorption, than the lower intestine. In contrast, the mucus layer covering epithelial cells is thicker in the ileum compared with upper small intestinal zones (Chikina and Vignjevic, 2021). However, it remains to be determined if there are shared or varied molecular identities of vessels of the small intestinal zones or other GI tract organs including the esophagus, stomach, cecum, colon, and anus.

In summary, intestinal villi are complex, highly stratified, organized structures that are crucial for intestinal homeostasis and function. Intestinal blood and lymphatic vessels are intertwined in this complex microenvironment and display both organ-specific and villus/crypt specialization necessary for gut nutrient absorption and homeostasis.

### Intestinal blood vasculature

Blood vessels are crucial to gut function and pervade all intestinal tissue layers. Intestinal blood flow is supplied through the superior and inferior mesenteric arteries that split into smaller mesenteric arteries and branch before perforating the mesothelial and muscle layers into the intestine. Larger submucosal arterioles run perpendicular to the villus/crypt axis and branch off to smaller arterioles which either feed the crypt or villus capillary networks. Villus precapillary arterioles are usually unbranched until reaching the villus tip where they feed into the cage-like villus capillary network. Although the villus and crypt capillary beds are distinct, they form a contiguous capillary network distributed from villus tip to crypt with the villus capillary bed being denser than the crypt vessels. However, post-capillary venules collect all capillary output in the villus and are unbranched until their connection with the submucosal venules that run parallel with arterioles. These venules follow the arterial network out of the muscle layer to the mesentery, where all intestinal-derived blood flows through the portal vein to the liver (Kvietys, 2013; Fig. 1, A and D).

Several features of the intestinal vasculature make it distinct. One, the intestine actively and drastically manipulates systemic blood flow. While at rest the intestinal vasculature receives around 20% of cardiac output, this can increase up to 60% postprandially (Granger et al., 2015). There is also intraintestinal control of blood flow; during feeding, up to 75% of blood is directed to the villus capillaries rather than submucosal vessels (Gore and Bohlen, 1977; Sababi and Holm, 1995). Conversely, strenuous exercise and other stress-related conditions strongly reduce intestinal blood flow (Granger et al., 2015), highlighting the gut’s systemic role in maintenance of vital organ perfusion. Intestinal blood flow is controlled by modulations in vascular tone and blood pressure via myogenic, metabolic (oxygen levels), chemical (adenosine and nitric oxide), and neural mechanisms (Granger et al., 2015). Therefore, villus/crypt blood flow decisions are likely mediated by regulation of distinct precapillary arterial resistance feeding the two capillary beds, positioning blood flow as a dynamic regulator of intestinal vessel function. Nevertheless, a comprehensive mechanistic model for regulating gut blood flow remains elusive at present.

The finger-like villus structure also contributes to another distinctive feature of these vessels. In most vascular beds, e.g., skin, arterioles and venules are physically separated by capillaries. However, in small intestinal villi a “counter-current” blood flow exists, such that the close proximity of arterioles and venules allows oxygen (O_2_) to “short-circuit” in the proximal villus zone (Fig. 1 D; Hallbäck et al., 1978; Jodal and Lundgren, 1986; Shepherd and Kiel, 1992). The counter-current blood flow, combined with O_2_ consumption by villus epithelial cells, results in a gradient of decreasing pO_2_ from the proximal to distal villus tip (Fig. 1, C and D; Granger et al., 2015). Villus hypoxia promotes high vascular endothelial growth factor A (VEGFA) expression from both epithelial cells and fibroblasts (Bernier-Latmani et al., 2022a, 2022b; Korsisaari et al., 2007), rendering the highly dense villus capillaries VEGFA dependent while displaying a sprouting and proliferating phenotype similar to that observed during developmental angiogenesis (Fig. 1 D; Bernier-Latmani et al., 2015; Bernier-Latmani et al., 2022a, 2022b; Bernier-Latmani and Petrova, 2016; Kamba et al., 2006; Karaman et al., 2022; Kido et al., 2022; Lee et al., 2007; Yang et al., 2013). Functionally, high VEGFA signaling induces formation of small pores in endothelial cells called fenestrations (Esser et al., 1998). VEGFA-induced endothelial cell fenestration renders these vessels highly permeable and able to rapidly uptake nutrients from enterocytes (Bernier-Latmani et al., 2022b; Kamba et al., 2006). Furthermore, the gut microbiota also enhances villus blood capillary density (Stappenbeck et al., 2002). These distinct characteristics show that gut blood vessels are uniquely adapted to the intestinal niche and specialized function.

In addition to the vessels of the villus/crypt axis, submucosal, and mesenteric regions, there is a distinct vasculature embedded in the gut muscle layer. These vessels are more dilated, sparse, and less branched than vessels in the villus/crypt zone (unpublished data). Recent single-cell RNA sequencing (RNAseq) data reveals that these vessels express genes for fat (*Cd36*, *Fabp5*) and water and glycerol (*Aqp1*, *Aqp7*) transport similar to those observed in muscle- and white adipose tissue–specific endothelial cells (Fan et al., 2021; Kalucka et al., 2020; Wiggins et al., 2023), suggesting they perform nutrient transport to support intestinal smooth muscle metabolism.

### Intestinal lymphatic vasculature

As for the blood vasculature, lymphatics are present throughout the gut. In general, the lymphatic vasculature can be categorized into two kinds of vessels: capillaries and collecting vessels. The former are permeable due to discontinuous “button” cell–cell junctions allowing the passive uptake of fluid and macromolecules and transmigration of immune cells. In contrast, the lymphatic collecting vessels are relatively impermeable due to continuous “zipper” cell–cell junctions, and, through alternating sets of associated SMCs and intraluminal valves, unidirectionally pump lymph through lymph nodes to venous circulation (Petrova and Koh, 2020). In the intestine proper, most lymphatic vessels in the villi and crypts are capillaries while collecting vessels are restricted to the mesentery (Fig. 1 E; Bernier-Latmani and Petrova, 2017). Intestinal lymph enters villus and submucosal lymphatic capillaries and flows to larger collecting vessels in the mesentery to the mesenteric lymph nodes through the cisterna chyli and thoracic duct and into blood circulation (Fig. 1, A and E). A recent study identified a separate lymphatic capillary network within the mesentery, which surveils the abdominal cavity and drains directly to the mediastinal lymph nodes (Redder et al., 2023, *Preprint*).

Lymphatic capillaries are differentially patterned along the villus/crypt axis. While submucosal crypt-associated lymphatics resemble their counterparts in other organs, the villus capillaries, also called lacteals, display filopodia and are reminiscent of sprouting lymphatic vessels during development (Fig. 1 E; Bernier-Latmani et al., 2015; Hong et al., 2020). Lacteal filopodia formation is in response to VEGFC production from villus fibroblasts, and VEGFC/VEGFR3 signaling is necessary to sustain these vessels (Bernier-Latmani et al., 2015; Hong et al., 2020; Nurmi et al., 2015; Suh et al., 2019; Zarkada et al., 2023). Furthermore, in contrast to submucosal lymphatic capillaries and filopodia-negative lacteals that display button junctions, filopodia-bearing lacteals have zippered junctions (Fig. 1 E; Bernier-Latmani et al., 2015; Hong et al., 2020; Zhang et al., 2018). Intestinal lymphatic endothelial cells isolated in recent single-cell RNAseq studies express common lymphatic markers such as *Prox1*, *Lyve1*, and *Pdpn* (Kalucka et al., 2020; Wiggins et al., 2023) and subdivide into four distinct clusters. Their functional relevance remains to be determined; however, a cluster with enrichment of interferon signaling (Wiggins et al., 2023) may be similar to the recently described *Ptx3*^*+*^ skin capillary lymphatic endothelial subset associated with immune cell recruitment (Petkova et al., 2023).

## Mechanisms of intestinal vessel nutrient uptake

Intestinal blood and lymphatic vessels are acknowledged as the route for systemic distribution of ingested nutrients, and the molecular mechanisms promoting these processes are now emerging. Recent studies have pointed to vascular mechanisms underlying this critical gut function.

### Blood vasculature

**Figure 2.**
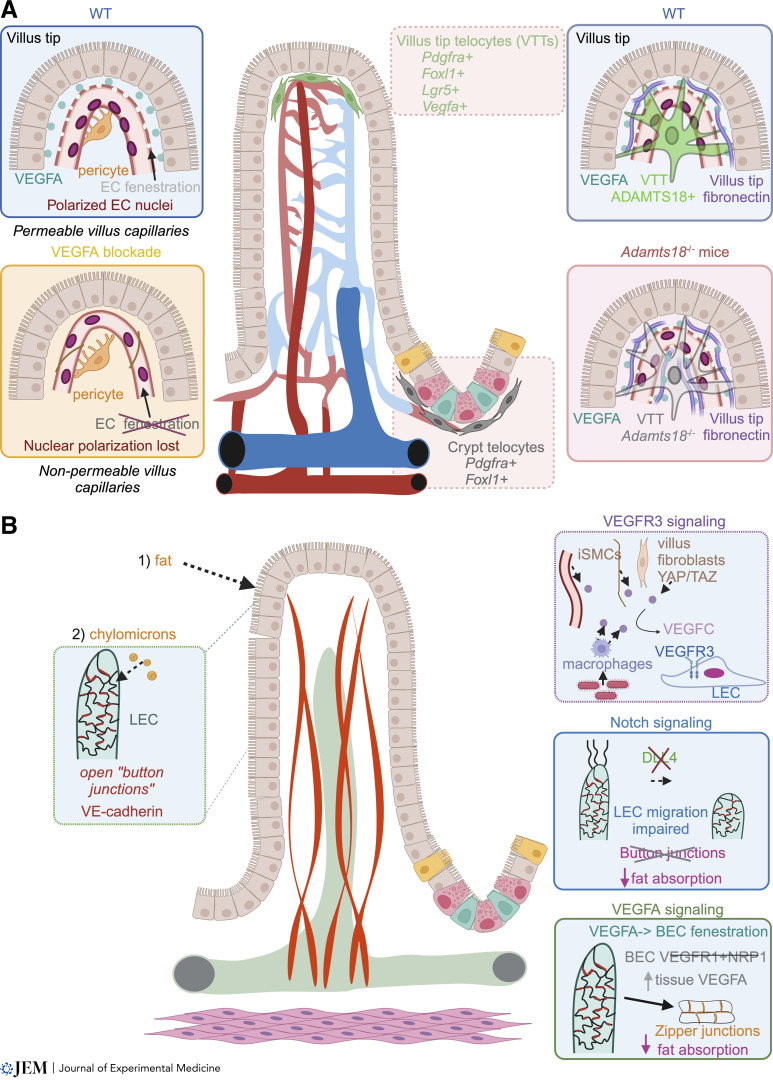
**Functional roles for intestinal vasculature in promoting nutrient absorption. (A)** Villus blood capillary endothelial cells display small pores, fenestrations, which promote rapid absorption of nutrients transported through the epithelium. High levels of villus VEGFA (from epithelial cells and fibroblasts) promote endothelial fenestration. VEGFA signaling is highest at the villus tip, and VEGFA protein and fenestrations are polarized to the epithelial side of endothelial cells (ECs). VEGFA blockade restricts fenestrations and EC nuclear polarization, making vessels less permeable. VTTs also promote polarized EC fenestration. These fibroblasts uniquely express the metalloprotease ADAMTS18, which degrades fibronectin, thus constraining VEGFA to the epithelial side of ECs. In the absence of VTTs or ADAMTS18, villus tip fibronectin accumulates and spreads bound VEGFA, thereby promoting widespread vessel fenestration and leakiness. **(B)** Lacteal cell–cell junction status controls dietary fat absorption. Enterocytes package dietary fat into lipoprotein particles called chylomicrons, which are size excluded from blood capillaries. Chylomicrons enter the lymphatic system through open button junctions between lacteal LECs. Increased VEGFC/VEGFR3 or VEGFA/VEGFR2 signaling or loss of Notch signaling leads to zippering of LEC junctions and decreased chylomicron absorption.

The highly permeable villus blood capillary network ensures highly efficient transport of carbohydrates, peptides, amino acids, and short-chain fatty acids from the gut lumen. These nutrients either diffuse across the epithelium or are actively transported paracellularly by villus enterocytes (van der Flier and Clevers, 2009). The dense capillary network is maintained through continuous VEGFA signaling, which also promotes endothelial cell fenestration (Bernier-Latmani et al., 2022a, 2022b; Kamba et al., 2006; Karaman et al., 2022; Kido et al., 2022; Lee et al., 2007; Yang et al., 2013). VEGFA signaling blockade reduces glucose absorption (Kamba et al., 2006) indicating that endothelial cell fenestration renders the villus capillaries permeable to ensure efficient nutrient uptake (Fig. 2 A).

As mentioned above, the villus tip is a distinct niche with many overlapping inputs: it is the most hypoxic zone of the villus and site of constant nutrient absorption and epithelial cell apoptosis (Fig. 1 C). Endothelial cells in villus tip capillaries are also highly organized and polarized as revealed by electron microscopy studies. Fenestrations are restricted to the epithelial side of villus tip endothelial cells while nuclei are located opposite on the villus core–facing side (Fig. 2 A; Casley-Smith, 1971; Casley-Smith et al., 1975; Milici and Bankston, 1981; Palay and Karlin, 1959). Moreover, villus tip endothelial cells are arranged into so-called “seamless” tubes such that the capillary lumen is surrounded by a single endothelial cell (Fig. 2 A; Wolff et al., 1972). This endothelial cell patterning is observed during developmental vessel anastomosis but is also found in other adult capillary beds such as the brain (Kotini et al., 2019; Wolff and Bär, 1972). In a recent work, we confirmed, through high-resolution confocal and 3D electron microscopy, the earlier observations of villus tip endothelial cell fenestration/nuclear polarization and seamless patterning (Bernier-Latmani et al., 2022b). In agreement with relatively low pO_2_ at the villus tip, endothelial VEGFA signaling was also highest in these villus tip endothelial cells and VEGFA protein deposition is limited to the epithelial side of vessels (Fig. 2 A). Blocking VEGFA signaling prevented the endothelial nucleus polarization and seamless endothelial cell phenotypes, suggesting an active mechanism to restrict VEGFA localization and promote the polarized nature of the villus tip niche (Bernier-Latmani et al., 2022b).

The mesenchymal intestinal villus core is separated from the epithelium by a syncytial layer of subepithelial fibroblasts, also called telocytes (Kaestner, 2019; McCarthy et al., 2020). These cells are defined by PDGFRA and *Foxl1* expression, but a subpopulation at the villus tip, villus tip telocytes (VTTs), also express the intestinal epithelial stem cell marker *Lgr5* (Bahar Halpern et al., 2020). VTTs are associated with villus tip polarized blood endothelial fenestrations and are necessary to maintain a spatially restricted VEGFA signaling domain as VTT depletion perturbed the villus tip endothelial cell arrangement and expanded VEGFA signaling throughout the villus blood vessels (Fig. 2 A; Bernier-Latmani et al., 2022b). Mechanistically, the secreted Zn^2+^-dependent metalloprotease ADAMTS18 is expressed uniquely by VTTs and is necessary to limit the spread of VEGFA and fenestrations to the epithelial side of villus tip capillaries through degradation of fibronectin, a main extracellular matrix component sequestering VEGFA. Functionally, *Adamts18*^*−/−*^ mice displayed increased circulating levels of certain amino acids highlighting the role of polarized endothelial villus tip phenotype in nutrient absorption (Fig. 2 A; Bernier-Latmani et al., 2022b). Therefore, an integrated communication network between VTTs and endothelial cells is necessary to promote maintenance of the specialized villus tip blood vessels.

Further molecular mechanisms promoting blood vessel–mediated nutrient absorption are currently unknown. However, spatial transcriptomic analysis of enterocytes revealed that the villus intestinal epithelium is functionally zonated such that specific nutrient transporters are positioned differentially from the proximal to distal villus (Moor et al., 2018). Whether nutrient specificity extends to the underlying absorptive vasculature or if separate villus zones are enriched in certain kinds of nutrients, which could directly impact endothelial cell signaling or phenotype, remains to be determined.

### Lymphatic vasculature

Villus-residing lacteals absorb fat packaged by enterocytes in the form of lipoprotein particles called chylomicrons. These particles diffuse through the villus core, and electron microscopy revealed they are taken up into lacteals intercellularly through open “flap valves” between lacteal lymphatic endothelial cells (LECs; Casley-Smith, 1962; Palay and Karlin, 1959; Sabesin and Frase, 1977; Tso and Balint, 1986). Flap valves are identified by button junctions that stain positive for LYVE1 between discontinuous vascular endothelial (VE)-cadherin junction staining and are present in lacteals (Fig. 2 B; Baluk et al., 2007; Bernier-Latmani et al., 2015; Hong et al., 2020; Zhang et al., 2018). VE-cadherin itself is necessary to maintain junction patterning of lymphatic vessels, including lacteals (Hägerling et al., 2018), and maintenance of button junctions is necessary to promote lacteal chylomicron absorption.

VEGFR signaling is one cue controlling lacteal junction status. Lymphatic VEGFR3 signaling is necessary for both developmental lacteal growth and maintenance in adults (Mäkinen et al., 2001; Kim et al., 2007; Tammela et al., 2008; Bernier-Latmani et al., 2015; Nurmi et al., 2015). The VEGFR3 ligand VEGFC is expressed by vascular and intestinal SMCs, macrophages, and a subset of villus fibroblasts (Hong et al., 2020; Nurmi et al., 2015; Suh et al., 2019). Microbiota exposure promotes VEGFC production by intestinal macrophages, whereas in fibroblasts, VEGFC is induced in response to YAP/TAZ signaling (Hong et al., 2020; Suh et al., 2019). Accordingly, hyperactivation of YAP/TAZ in PDGFRβ^+^ intestinal fibroblasts leads to extreme lacteal overgrowth, junction zippering, and loss of fat absorption capacity (Fig. 2 B; Hong et al., 2020). Similarly, Notch signaling downstream of VEGFC/VEGFR3 signaling is also necessary for lacteal length maintenance in adult mice. Lymphatic-specific ablation of the Notch ligand DLL4 inhibited lacteal LEC migration and formation of button junctions, leading to impaired dietary fat absorption (Fig. 2 B; Bernier-Latmani et al., 2015). Lymphatic-specific deletion of the adrenomedullin receptor CALCRL also leads to a loss of Notch signaling and lacteal defects (Davis et al., 2017; Hoopes et al., 2012).

Lacteal button junctions are sensitive to incoming chylomicrons and VEGFA, produced at the villus tip to promote blood vessel fenestrations (Bernier-Latmani et al., 2022a, 2022b; Korsisaari et al., 2007). Notably, chylomicron-derived lipids facilitate LEC junction opening via ROCK-dependent contraction of junction-anchored stress fibers (Zarkada et al., 2023). On the contrary, excessive levels of VEGFA lead to junction zippering and loss of fat absorption, as observed upon blood endothelial cell-specific loss of the VEGFA sinks VEGFR1 and NRP1 (Fig. 2 B; Zhang et al., 2018). VEGFA may signal through VEGFR2/3 heterodimers rather than solely VEGFR2 as independent lymphatic-specific ablation of the two receptors blocks VEGFA-mediated lacteal junction zippering (Zarkada et al., 2023), although long-term lymphatic VEGFR3 deletion leads to lacteal zippering (Jannaway et al., 2023). Mechanistically, increased signaling of VEGFR2/3 heterodimers phosphorylates PI3K/Akt leading to lacteal zippering by inhibiting ROCK, which promotes stabilization of the LEC cytoskeleton to maintain button junctions (Zarkada et al., 2023; Zhang et al., 2018). Therefore, a combination of lipid-driven ROCK activation and lacteal VEGFR2/3 signaling is crucial for efficient fat absorption.

Lymphatic interaction with the autonomic nervous system and villus SMCs also promotes fat absorption. Lacteals are ensheathed in villus SMCs (Bernier-Latmani et al., 2015; Bernier-Latmani and Petrova, 2016; Choe et al., 2015), which pump the vessels to promote chylomicron absorption. Pumping is mediated at least in part by the autonomic nervous system as lacteals are in close contact with enteric nerves and autonomic nervous blockade inhibits both pumping and fat absorption (Bachmann et al., 2019; Choe et al., 2015).

Lacteal function also requires proper developmental patterning of SMCs. Villus SMCs express the transcription factor PITX2, which controls left/right gut folding symmetry thus promoting proper blood vessel patterning and prevention of intestinal ischemia (Mahadevan et al., 2014). Interestingly, the *Pitx2 ASE* promoter, driving asymmetric expression of *Pitx2*, is necessary for villus SMC precursor expansion, development into lacteal-associated SMCs, and lacteal sprouting. *Pitx2 ASE* mutant pups display liver steatosis, suggesting that under pathological conditions normally size-excluded chylomicrons can enter the villus blood capillaries and reach the liver via the portal vein (Hu et al., 2021). Vice versa, lacteal-derived DLL4 may contribute to development of lacteal-associated SMCs by activating NOTCH3 in PDGFRA^+^ villus fibroblasts (Sanketi et al., 2023, *Preprint*).

## Nutrient absorption–independent roles for gut vasculature

### Maintenance of the intestinal stem cell niche

**Figure 3.**
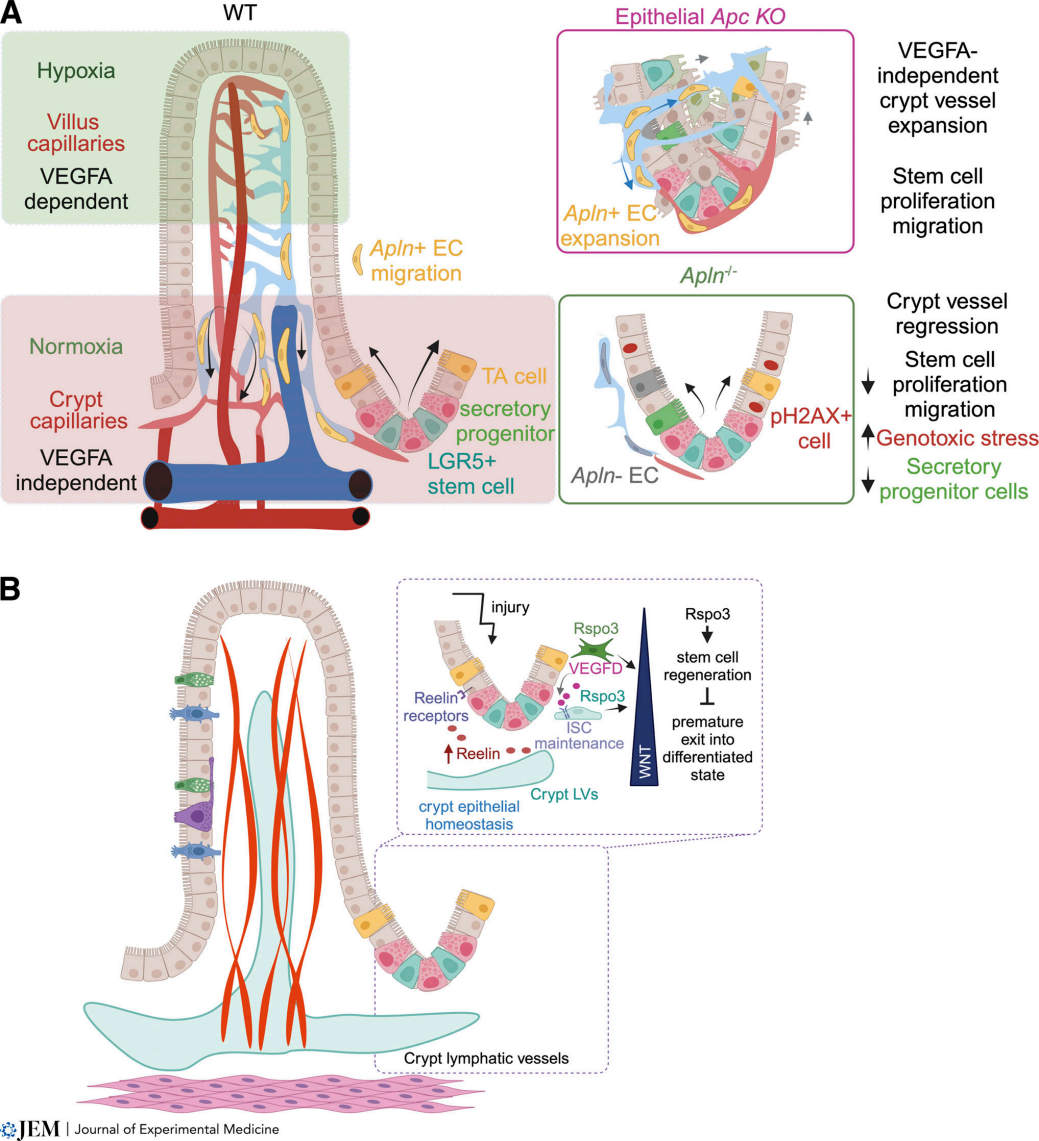
**Role of intestinal vasculatures in maintaining epithelial homeostasis. (A)** Intestinal villi are hypoxic, leading to VEGFA expression and the presence of VEGFA-dependent blood capillaries. In contrast, crypts are normoxic, O_2_ is necessary for stem cell maintenance, and blood vessels are VEGFA independent. Rather, crypt vessel expansion depends on apelin (Apln) signaling to promote intravessel endothelial cell (EC) migration from the villus to the crypt. During pathological crypt expansion, e.g., following epithelial *APC* loss-of-function (initiating mutation in colon cancer), blood capillaries expand in a VEGFA-independent manner, allowing stem cell proliferation and migration. In the absence of Apln, crypt vessels lack endothelial cells to maintain vessel patency resulting in crypt hypoxia and epithelial progenitor cell death. **(B)** Crypt lymphatics promote emergency stem cell maintenance. The Wnt signaling potentiator RSPO3 is produced by crypt lymphatics and fibroblasts, where it promotes stem cell maintenance, especially during intestinal injury. The secreted protein reelin is also expressed by crypt lymphatics and contributes to epithelial regrowth after injury by binding its receptors VLDLR, ITGB1, and LRP8. ISC, intestinal stem cell.

As opposed to the dense VEGFA-dependent villus blood capillaries, crypt capillaries are more sparse, less branched, and VEGFA independent. This property enables them to maintain a normoxic niche around intestinal stem cells (ISCs) and preserve ISC function even during VEGFA signaling blockade (Fig. 3 A; Bernier-Latmani et al., 2022a). Yet, crypt-associated blood capillaries are highly plastic and rapidly expand when the number of intestinal stem/progenitor cells is acutely increased upon epithelial-specific *Apc* ablation to maintain epithelial normoxia. Mechanistically, such vascular expansion is proliferation-independent and is driven by increased migration of apelin^+^ villus endothelial cells to expanding crypt vessels (Fig. 3 A). Loss of endothelial cell migration and crypt vessel patency in *Apln*^−/−^ mice resulted in decreased epithelial cell proliferation, increased genotoxic stress, and depletion of secretory progenitor cells in the normal gut and even more pronounced loss of stem cells in intestinal tumors (Fig. 3 A; Bernier-Latmani et al., 2022a). These observations in adult mice and tumors agree with models of developmental endothelial cell movement where constant migration of venous endothelial cells through the capillary bed supplies vascular expansion (Lee et al., 2021; Pitulescu et al., 2017; Xu et al., 2014) and highlight a novel vascular accrual mechanism for maintenance of the adult ISC niche under normal and pathological conditions.

Transgenic mice with inducible epithelial overexpression of VEGFA or VEGFA trap sVEGFR1 displayed larger and smaller villi, respectively. Furthermore, there were distinct effects on crypt epithelial cells with increased VEGFA-enhancing upper crypt proliferation and decreased VEGFA signaling limiting proliferation to the crypt bottom and augmenting the number of Paneth cells (Schlieve et al., 2016). Therefore, differential patterning and signaling along the villus/crypt axis blood vasculature directly contribute to the homeostasis of intestinal epithelial cells.

A recent trio of papers showed an important paracrine role for crypt lymphatics in ISC maintenance, especially during regeneration after injury (Goto et al., 2022; Niec et al., 2022; Palikuqi et al., 2022). Wnt signaling in intestinal stem/progenitor cells is secured by multiple cellular sources, such as Paneth cells, pericryptal fibroblasts, and macrophages, which produce Wnt ligands and potentiators such as R-spondins (RSPOs; Brügger and Basler, 2023; Gehart and Clevers, 2019; Sylvestre et al., 2023). Bulk RNAseq revealed that in addition to the above cell types, intestinal lymphatics also express *Wnt2* and *Rspo3* (Ogasawara et al., 2018), and this was further confirmed by single-cell RNAseq (Goto et al., 2022; Niec et al., 2022; Palikuqi et al., 2022). Lymphatic RSPO3 functionally contributes to crypt epithelial cell proliferation during pathological regeneration in response to injury, such as irradiation or inflammation (Goto et al., 2022; Palikuqi et al., 2022; Tan et al., 2023). However, overlapping expression of *Rspo3* by fibroblasts and lymphatics likely evolved to ensure crypt Wnt signaling as only simultaneous deletion in both cell types caused a significant decrease in crypt cell proliferation (Fig. 3 B; Goto et al., 2022).

In addition to RSPO3, intestinal lymphatics may contribute reelin signaling to the intestinal crypt. Reelin is a secreted protein highly expressed in neurons and lymphatic capillaries (Herz and Chen, 2006; Lutter et al., 2012), and lymphatic-derived reelin contributes to heart regeneration after myocardial infarction (Liu et al., 2020). *Reln* is highly expressed in intestinal submucosal lymphatics near crypts (Goto et al., 2022; Niec et al., 2022; Palikuqi et al., 2022). It contributes to crypt epithelial homeostasis as mice with germline *Reln* mutations (Reeler mice; D’Arcangelo et al., 1995) display decreased crypt epithelial cell proliferation and Paneth cell numbers (García-Miranda et al., 2013). In contrast, lymphatic-specific *Reln* deletion results in increased crypt epithelial cell proliferation, but maintenance of ISCs (Niec et al., 2022), suggesting that lymphatic-derived Reelin may act indirectly to promote crypt epithelial homeostasis (Fig. 3 B). While the Reelin receptor *Itgb1* is widely expressed in intestinal epithelial cells, two other Reelin receptors, *Vldlr* and *Lrp8*, are limited to secretory progenitor, Paneth, goblet, and enteroendocrine cells (Haber et al., 2017; Niec et al., 2022). Therefore, given the results of decreased Paneth cell numbers and increased goblet cell numbers in germline Reeler mice (García-Miranda et al., 2013), lymphatic-to-epithelial signaling could be acting on crypt secretory progenitor cells and alter differentiated epithelial cell fate decisions (Fig. 3 B).

### Gut immune cell recruitment

**Figure 4.**
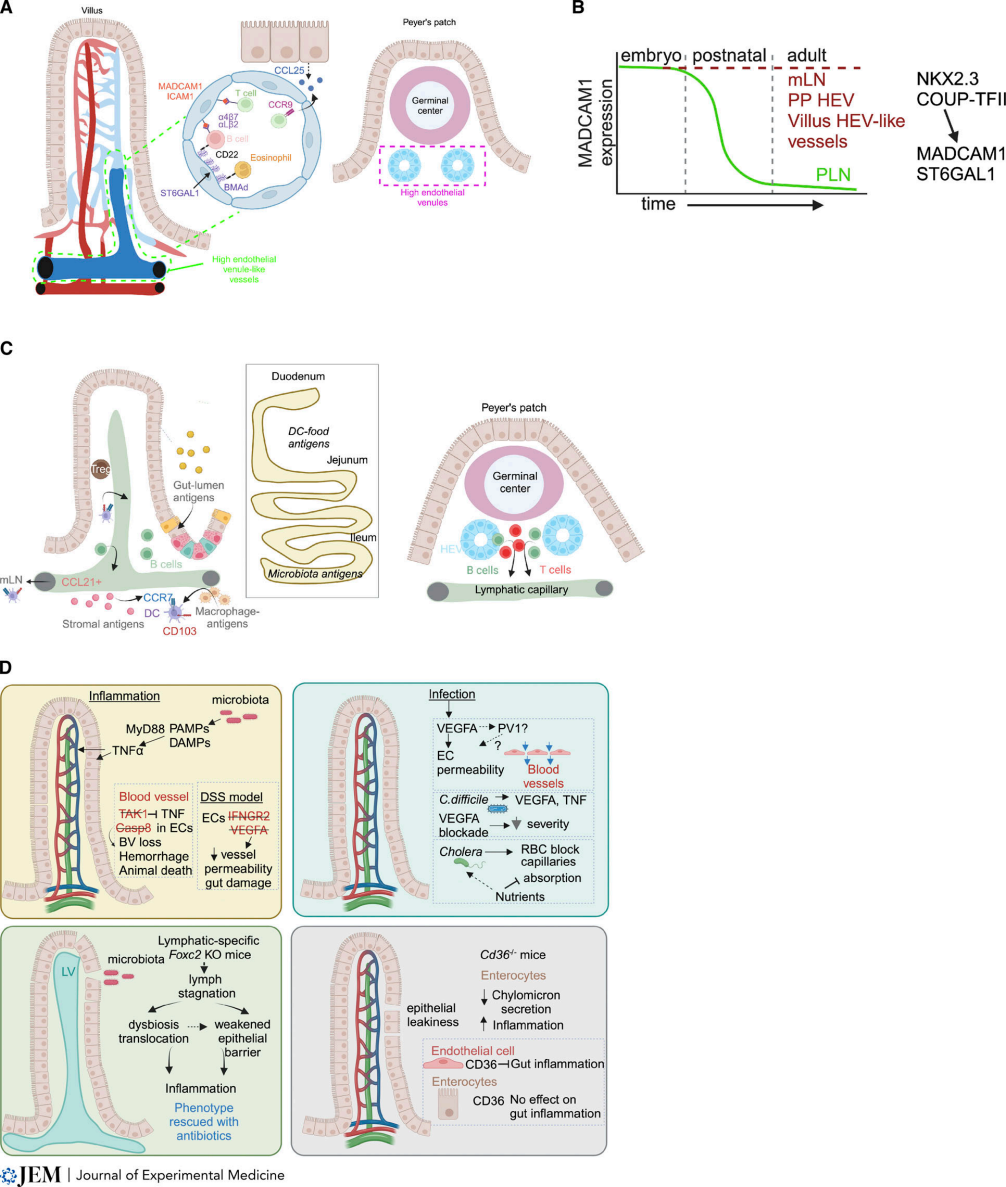
**Roles for the intestinal vasculatures in immune cell trafficking and response to gut microbiota. (A)** Immune cell transport by intestinal blood vessels. Post-capillary HEV-like vessels present in each small intestinal villus. HEVs in PPs, GALT, express addressin proteins that enable gut-specific immune cell recruitment. HEVs and villus HEV-like vessels express MADCAM1 and ICAM1, which bind α4β7 and αLβ2 on lymphocytes, respectively. ST6GAL1 codes for a glycan siaylating protein, which produces the BMAd. Additionally, enterocytes express CCL25 chemokine promoting extravasation of CCR9-expressing lymphocytes in the gut. **(B)** Developmental control of mucosal addressins. Embryonic HEVs of PLN and MLN express MADCAM1, which is restricted postnatally to the GI tract HEVs and HEV-like villus vessels. This is mediated in part by cooperation between the transcription factors NKX2.3 and COUP-TFII, which also promote expression of the gene encoding the BMAd-generating enzyme ST6GAL1. **(C)** Immune cell transport by intestinal lymphatics. Lymphatic capillaries recruit CCR7^+^ immune cells through secretion of CCL21. A major population that is trafficked from the gut are CD103^+^ DCs, which carry antigens from a variety of sources. Most B cells are recirculated via PP lymphatic vessels (Reboldi and Cyster, 2016). While DC-carried food antigens are more prevalent in the duodenum, microbiota-derived antigens are found in ileum DCs. Treg, regulatory T cell. **(D)** Intestinal vessels display specialized mechanisms to resist microbiota-driven inflammation. These include resistance to TNF signaling (TAK1, CASP8), modulation of permeability in response to infection, and maintenance of lymph outflow of immune cells and chylomicrons. PAMP, pathogen-associated molecular pattern; DAMP, damage-associated molecular pattern; EC, endothelial cell.

The adult intestine is one of the most immune cell–rich organs. This high density is maintained through constant gut-tropic immune cell recruitment (Mowat and Agace, 2014) by high endothelial venules (HEVs) of Peyer’s patches (PPs) and venules of intestinal villi. Despite their overall similar structural organization and function in recruitment of naïve lymphocytes, PP and non-gut-associated peripheral lymph node (PLN; Habtezion et al., 2016) high endothelial cells (HECs) also display substantial differences. For example, adult PLN, but not PP HECs, express a unique complex of heavily sialyated glycoproteins that constitute the peripheral lymph node addressin (PNAd; Girard et al., 2012), a ligand for the L-selectin receptor which initiates immune cell rolling and tethering to the endothelium. Accordingly, PLN HECs also express PNAd-constructing proteins, including *Chst4* and *Fut7* (Lee et al., 2014). In contrast, PP HECs produce the mucosal addressin protein MADCAM1, which acts as a ligand for integrin α4β7 expressed on gut-tropic immune cells (Berlin et al., 1993, 1995). Integrin α4β7 expression is “imprinted” on naïve lymphocytes by dendritic cells possessing intestinal antigen and displaying elevated retinoic acid production (Stagg, 2018). Retinoic acid signaling then induces expression of integrin α4β7 on lymphocytes, which directs them to the gut. To reinforce efficient gut extravasation, gut-tropic lymphocytes express CCR9 and follow gradients of small intestinal epithelial cell–derived CCL25 (Fig. 4 A; Rivera and Lennon-Duménil, 2023).

In addition to the PPs, immune cells extravasate into small intestinal villi. Each villus contains an HEV-like MADCAM1^+^ICAM1^+^ venule from where α4β7 and αLβ2 expressing lymphocytes are recruited. ST6GAL1, a glycan sialylating enzyme expressed highly in gut-associated lymphoid tissue (GALT) HEVs (Lee et al., 2014) and a subset of villus vessels (Dinh et al., 2022), promotes formation of the “B cell–specific mucosal vascular addressin” (BMAd). The BMAd binds CD22, a surface protein expressed on B cells and intestine-specific eosinophils (Habtezion et al., 2016; Wen et al., 2012), and promotes gut-specific extravasation (Fig. 4 A; Lee et al., 2014). Therefore, intestinal-specific vascular “addresses” combined with immune cell imprinting are critical for recruitment of enterotropic lymphocytes and gut immunosurveillance.

Gut-specific expression of MADCAM1 is regulated dynamically during development. In the embryonic and early postnatal period, MADCAM1 is widely expressed in HEVs of both peripheral and mesenteric LNs, PPs, and intestinal villus capillaries (Iizuka et al., 2000; Mebius et al., 1996; Salmi et al., 2001). However, its expression is restricted rapidly after birth to a subset of LN lymphatic vessels, GALT HEVs, and villus/crypt HEV-like venules (Fig. 4 B; Arroz-Madeira et al., 2023; Salmi et al., 2001). In early postnatal and adult mice, MADCAM1 expression is promoted by the transcription factors NKX2-3 and COUP-TFII (Dinh et al., 2022; Kellermayer et al., 2014; Pabst et al., 2000; Pabst et al., 1999; Wang et al., 2000). NKX2-3 is highly expressed in derivatives of visceral mesoderm, such as gut endothelial and mesenchymal cells (Fig. 4 B; Pabst et al., 1997; Wang et al., 2000), and is necessary for proper intestine development. *Nkx2-3*^*−/−*^ mice display severely decreased villus size, a dearth of villus capillaries and, importantly, lack MADCAM1-expressing vessels (Kellermayer et al., 2014; Pabst et al., 2000; Pabst et al., 1999; Wang et al., 2000). Mechanistically, NKX2-3 heterodimerizes with the venous master regulator COUP-TFII to promote intestinal *Madcam1* and *St6gal1* expression via cooperative binding to conserved composite regulatory elements (Dinh et al., 2022). Accordingly, endothelial COUP-TFII overexpression or ablation is sufficient to increase or decrease intestinal MADCAM1 and BMAd (Dinh et al., 2022), highlighting the importance of NKX2-3 and COUP-TFII cooperation for the intestinal-specific endothelial identity (Fig. 4 B). What promotes persistence of gut-specific activity of *Nkx2-3* and early postnatal loss of MADCAM1 expression elsewhere remains unclear. However, antibiotic treatment reduces MADCAM1 in ileal crypt/villus and GALT vessels, suggesting a role of the microbiota in MADCAM1 expression (Fidelle et al., 2023).

### Lymphatic vessel immune cell transport

Tolerance to gut-derived antigens is maintained by constant migration of immune cells from the gut to the mesenteric LN (MLN), and intestinal lymphatic vessels are critical for this function. Ablation of gut lymphatics causes rapid death of mice due to massive intestinal inflammation (Jang et al., 2013), likely due to loss of tolerance, showing the importance of this function. A recent review highlighted mechanisms of PP lymphatic function (Arroz-Madeira et al., 2023); therefore, below we will focus on lymphatic immune trafficking along the crypt/villus unit.

Dendritic cells (DCs) are the predominant population mediating intestinal immune tolerance to food antigens (Mowat and Agace, 2014; Pabst and Mowat, 2012). DCs capture antigen directly in the intestinal stroma from resident macrophages or directly from the gut lumen (Bogunovic et al., 2009; Cerovic et al., 2014; Chang et al., 2013; Farache et al., 2013; McDole et al., 2012; Schulz et al., 2009). Migratory DCs mostly express CD103 as well as the chemokine receptor CCR7, which promotes migration toward the CCL21^+^ lymphatic capillaries and to the MLN (Fig. 4 C; Bogunovic et al., 2009; Cerovic et al., 2013; Johansson-Lindbom et al., 2005; Schulz et al., 2009; Worbs et al., 2006). As mentioned above, intestine-derived DCs imprint lymphocytes with gut tropism through retinoic acid signaling (Agace and Persson, 2012), and further TGFβ signaling promotes differentiation of regulatory T cells necessary for immunosuppression upon migration back to the intestine through the blood vasculature (Fig. 4 C; Cassani et al., 2011; Coombes et al., 2007; Cording et al., 2014; Hadis et al., 2011; Jaensson-Gyllenbäck et al., 2011; Sun et al., 2007). Food digestion and ingestion take place in the upper small intestine, therefore DC-carried food antigens are more prevalent in the duodenum while microbiota-derived antigens are found in the DCs derived from the ileum. This gradient of antigen content along the length of the small intestine promotes tolerogenic and immune responses in the proximal and distal small intestine, respectively (Fig. 4 C; Esterházy et al., 2019; Houston et al., 2016). In addition, duodenal DCs also promote tolerogenic responses in LNs that share drainage with the more inflammatory pancreas (Brown et al., 2023). Whether lymphatic vessel immune cell trafficking is altered among intestinal compartments or other organs is yet to be studied.

## Intestinal vessel pathology

Defects in both intestinal blood and lymphatic vessels are an acute cause of several diseases, underlining their importance in human health. Molecular mechanisms promoting intestinal vessel dysfunction are still emerging, highlighting the need for further study to develop novel targeted therapies.

The most common pathology associated with gut blood vessels is intestinal ischemia. Decreased intestinal blood flow through vessel damage or occlusion can be caused by a plethora of diseases, including heart disease, atherosclerosis, dehydration, infection, autoimmune disease, stimulant use, and thrombophilia (Clair and Beach, 2016). Intestinal ischemia causes acute villus tip epithelial cell death, progressively leading to villus shortening and villus vessel loss (Haglund, 1994). Necrotizing enterocolitis (NEC) is a common cause of mortality in premature babies and its etiology is likely multifactorial, including abnormal gut microbiota, high levels of inflammation, and intestinal ischemia (Neu and Walker, 2011). Inflammation and decreased VEGFA signaling may drive gut ischemia in NEC patients by limiting developmental intestinal angiogenesis (Bowker et al., 2018; Yan et al., 2016, 2019). Regrowth of blood vasculature during post-ischemic reperfusion may require VEGFA, but also endothelial expression of FOXC1 and FOXC2 transcription factors (Tan et al., 2023).

The intestinal vasculature is specialized to traffic specific immune cells in and out of the intestine for immunosurveillance of gut lumen contents. In turn, the microbiota and related inflammation also can directly impact the intestinal vasculature, raising the question of molecular mechanisms underlying resistance of gut endothelial cells to inflammation and constant exposure to pathogen-associated molecular patterns and damage-associated molecular patterns. The microbiota promotes constant TNF signaling in normal gut villus vessels, which display specialized resistance to TNF-mediated cell death (Kalliolias and Ivashkiv, 2016). Ablation of TAK1, a negative regulator of TNF signaling, in endothelial cells leads to rapid intestinal vessel loss, hemorrhaging, and animal death (Houston et al., 2016; Naito et al., 2019). Likewise, loss of endothelial CASP8, which prevents TNF-driven necroptosis (van Loo and Bertrand, 2023), leads to hemorrhaging and animal death (Fig. 4 D; Bader et al., 2023; Tisch et al., 2022). Though pan-endothelial *Casp8* depletion led to blood vessel defects, mice with blood endothelial cell–specific *Casp8* ablation were normal (Tisch et al., 2022), suggesting that repression of TNF-dependent necroptosis in lymphatics is especially crucial for gut homeostasis. Therefore, there are TNF protection mechanisms for intestinal endothelial cells, and future work will clarify the contribution of lymphatic and blood vessels.

One effect of intestinal infection or inflammation is increased epithelial gut permeability causing lumen contents to be in direct contact with the underlying stromal cells (Thoo et al., 2019). As mentioned above, VEGFA is a potent inducer of endothelial permeability (Claesson-Welsh, 2015), and its expression is increased following gut infection, leading to increased vessel permeability. *Clostridium difficile* infection rapidly promotes VEGFA and TNF expression, and disease severity was reduced with VEGFA signaling blockade (Fig. 4 D; Huang et al., 2019). In addition, in the DSS inflammation model, ablation of endothelial IFNGR2 and VEGFA signaling blockade ameliorated gut damage and decreased vessel permeability (Fig. 4 D; Langer et al., 2019). A subset of small high-density lipoproteins (HDL-C) produced by the intestine are transported via the portal vein to the liver rather than being absorbed by lymphatics. This transport is functionally important as HDL-C binds blood LPS to prevent inflammatory activation in the liver (Han et al., 2021).

Gut infection also produces other changes in intestinal vessels. Cholera infection causes red blood cell blockage of ileum capillaries to shunt host nutrients to the bacteria (Rivera-Chávez and Mekalanos, 2019). Furthermore, helminth worms physically break vessels when they penetrate the gut lining and use the blood as feed (Fig. 4 D; Gentile and King, 2018).

Inflammatory bowel diseases (IBDs), comprised of ulcerative colitis and Crohn’s disease, are the result of dysfunctional intestinal immune responses causing loss of the epithelial barrier and gut microbiota dysbiosis (Graham and Xavier, 2020). Lymphatic vessel dysfunction has long been implicated in Crohn’s disease (Crohn and Janowitz, 1954) and mice with lymphatic-specific ablation of *Foxc2* lose lymphatic valve function leading to an inability to transport gut-derived lymph (González-Loyola et al., 2021; Petrova et al., 2004; Sabine et al., 2012; Sabine et al., 2015). In turn, stagnation of intestinal lymph increases gut permeability, leading to gut dysbiosis and peritoneal inflammation followed by pleural effusion, which are all rescued with antibiotic treatment (Fig. 4 D; González-Loyola et al., 2021). In addition, continuous TNF-driven gut inflammation leads to lymphatic dysfunction and formation of mesenteric lymphoid structures, which may be a feed-forward mechanism in Crohn’s disease pathology (Czepielewski et al., 2021; Randolph et al., 2016).

The fat transporter/scavenger receptor protein CD36 (Chen et al., 2022) also promotes intestinal barrier function. Enterocytes in *Cd36*^*−/−*^ mice absorb fat from the gut lumen normally; however, display attenuated ability to secrete chylomicrons to the villus stroma (Drover et al., 2005; Goudriaan et al., 2005; Nauli et al., 2006). *Cd36*^*−/−*^ mice also display increased inflammation and epithelial leakiness; however, a comparison of epithelial and pan-endothelial *Cd36* deletion showed endothelial CD36 is necessary to suppress gut inflammation, while epithelial CD36 was dispensable (Fig. 4 D; Cifarelli et al., 2017). Lymphatic-specific deletion of CD36 led to disturbances in lacteal length and cell–cell junctions as well as leakiness in mesenteric lymphatic collecting vessels (Cifarelli et al., 2021). Compared with blood endothelial cells and collecting vessel LECs, lacteal LECs express relatively low levels of *Cd36* (Cifarelli et al., 2021; González-Loyola et al., 2021; Kalucka et al., 2020), suggesting that the lacteal phenotype maybe secondary to downstream collecting vessel defects or loss of lymphatic flow. Furthermore, Kawasaki disease, a childhood vasculitis leading to aneurysms of the coronary artery, is thought to arise from gut barrier dysfunction. Indeed, in mouse models of this disease, accumulation of gut-derived IgA antibodies is detected in coronary arteries of infected mice dependent on IL1β epithelial cell signaling (Noval Rivas et al., 2019).

Epithelial barrier loss and lymphatic dysfunction also manifest in protein-losing enteropathy, which is uncontrolled loss of circulating protein into the intestinal lumen, in the absence of liver or kidney disease, causing systemic hypoproteinemia (Ozen and Lenardo, 2023). This syndrome is observed in IBD patients and patients with hereditary intestinal lymphangiectasia such as in Hennekam syndrome (Bernier-Latmani and Petrova, 2017; Ozen and Lenardo, 2023).

In pathological conditions, villus vessels may serve as entry points for dissemination of microorganisms or inflammatory substances (bacteria, fat, LPS) after penetration of the epithelial and sub-epithelial fibroblast layers (Bertocchi et al., 2021; Carloni et al., 2021; Mouries et al., 2019; Spadoni et al., 2015). The proposed mechanism of such gut/vascular barrier (GVB) disruption involves upregulation of the endothelial-specific protein PLVAP (PV1), analogous to PLVAP induction upon loss of brain endothelial β-catenin leading to loss of blood–brain barrier integrity and brain vascular leakage (Liebner et al., 2008).

Recent single-cell RNAseq data of intestinal endothelial cells show high homeostatic expression of *Plvap*, especially in capillaries (Kalucka et al., 2020; Tan et al., 2023; Wiggins et al., 2023), consistent with its induction in response to VEGFA (Strickland et al., 2005) and its role as a functional component of fenestrae diaphragms, which are readily observed in gut capillaries (Bernier-Latmani et al., 2022b; Casley-Smith, 1971; Casley-Smith et al., 1975; Milici and Bankston, 1981; Palay and Karlin, 1959; Stan et al., 2012). These diaphragms promote vessel barrier function as mice with germline and endothelial *Plvap* ablation and patients with *PLVAP* mutations display leaky intestinal vessels and edema (Elkadri et al., 2015; Stan et al., 2012). Therefore, the mechanistic role of PLVAP in the GVB remains unclear.

## Emerging topics

### Sex-specific differences

Mice display sexual dimorphism of dietary fat transport. In general, male rodents transport ingested fat more efficiently than females (Yang et al., 2014). However, analysis of sexual dimorphism in intestinal fat transport ability showed a subset of female rats was able to transport fat at similar rates to males (Liu et al., 2021). Interestingly, ovariectomized female rats displayed fat transport rates similar to males, but lymphatic fat clearance could be reduced by treating the rats with either estradiol alone or an estradiol/progesterone mix, suggesting that ovarian hormones suppress lymphatic fat uptake (Liu et al., 2021). As mentioned above, lacteal junction zippering was observed following increased VEGFA signaling (Zarkada et al., 2023; Zhang et al., 2018), and ovariectomized rats under estradiol or an estradiol/progesterone treatment significantly enhanced gut *Vegfa* expression, though lacteal junctions were not analyzed (Liu et al., 2021). These observations suggest an epithelial-to-lymphatic communication to prevent chylomicron escape from villi during estrus. A driving force for this phenomenon could be the drastic changes in small intestinal morphology during pregnancy and lactation. In rodents, villi are significantly longer in pregnant compared to nulliparous dams, and villus capillary density increases during gestation (Meyer and Caton, 2016). Therefore, induction of *Vegfa* expression and intestinal fat retention could serve to increase vessel density to enhance energy reserves and oxygen to make a “metabolic nest” for villus expansion during pregnancy. However, despite this long-known female-specific intestinal transformation phenomenon, the mechanistic details of vascular expansion during pregnancy and molecular gender differences of the gut vasculature are not currently known.

### Circadian rhythms

The 24-h period of clock gene cycling in the hypothalamic suprachiasmatic nucleus dictates the activity/rest cycles of mammals and thereby controls feeding and sleep patterns (Takahashi, 2017). Circadian rhythms are driven by oscillating expression of the “core” clock genes controlled by the transcription factor BMAL1 (Koronowski and Sassone-Corsi, 2021). In the gut, circadian feeding patterns impart rhythmic changes in lumen contents, intestinal motility, and hormone levels (Segers and Depoortere, 2021). The gut microbiota is critical not only for Toll-like receptor signaling-dependent host circadian gene expression but also for the intestinal microbiota abundance, diversity, and positioning, which is regulated in a circadian manner (Litichevskiy and Thaiss, 2022).

Endothelial cells also display circadian expression of the core clock genes, and endothelial-specific *Bmal1* ablation decreases endothelial cell proliferation and impedes angiogenesis (Astone et al., 2023). Germline *Bmal1*^*−/−*^ adult mice display no gross intestinal vessel phenotype (unpublished data); whether intestinal vascular function is regulated by circadian rhythms remains to be determined. However, lacteals and submucosal lymphatic vessels display circadian expression of LYVE1 and other cell adhesion molecules, similar to lymphatic capillaries from other organs. Lymphatic-specific *Bmal1* deletion reduces DC migration into dermal lymphatic capillaries by reducing CCL21 expression (Holtkamp et al., 2021). Whether a similar mechanism exists for lacteals remains to be determined. Additionally, PLN HEV ICAM1 expression during inflammation is dependent on intact endothelial BMAL1 (Ince et al., 2023); however, whether gut tropic immune cell extravasation depends on circadian rhythms of HEV-like villus venules also remains unexplored.

### Gut-to-“X” (G2X) organ signaling

There has been intense study of gut communication with other organs: gut-to-brain, gut-to-liver, etc. (the G2X axes; Cryan et al., 2019; Dang and Marsland, 2019; Pabst et al., 2023). Hormones, microbial metabolites and lipids, and small peptide bioactive molecules are all distributed from the gut and are mediators of cross-organ communication (Lavelle and Sokol, 2020). Although much of this G2X communication has been attributed to the nervous system, vessels, as a conduit for systemic distribution of nutrients, are also integral to delivery of gut-derived molecules (Cryan et al., 2019). Therefore, intestinal vessels are a crucial link in the G2X axes. Distinguishing active and passive mechanisms for intestinal vessel G2X signaling will be crucial for defining novel systemic roles of gut vessels that may be exploited therapeutically.

## Concluding remarks

Electron microscopy studies from the past decades provided insights into the distinct patterning and unique properties of the intestinal vasculature. Advances in genetic mouse models, imaging, and single-cell genomic technologies have now enabled understanding of the molecular mechanisms underpinning intestinal vessel form and function. In addition, intense research of other intestinal cell types allows identification of novel intestinal vessel functions in the broader context of signaling and exchange in the functional villus/crypt unit. Therefore, future work will further unravel how intestinal vessels are a distinct but integral part of the intestinal cellular ecosystem.
